# Remote sensing image dehazing using generative adversarial network with texture and color space enhancement

**DOI:** 10.1038/s41598-024-63259-6

**Published:** 2024-05-29

**Authors:** Helin Shen, Tie Zhong, Yanfei Jia, Chunming Wu

**Affiliations:** 1 Key Laboratory of Modern Power System Simulation and Control and Renewable Energy Technology (Ministry of Education), Department of Communication Engineering, College of Electric Engineering, Northeast Electric Power University, Jilin, 132012 China; 2 College of Electric Power Engineering, Beihua Univesity, Jilin, 132012 China

**Keywords:** Remote sensing, Haze removal, Deep learning, Generative adversarial network (GAN), Computational neuroscience, Network models, Computational biology and bioinformatics, Image processing

## Abstract

Remote sensing is gradually playing an important role in the detection of ground information. However, the quality of remote-sensing images has always suffered from unexpected natural conditions, such as intense haze phenomenon. Recently, convolutional neural networks (CNNs) have been applied to deal with dehazing problems, and some important findings have been obtained. Unfortunately, the performance of these classical CNN-based methods still needs further enhancement owing to their limited feature extraction capability. As a critical branch of CNNs, the generative adversarial network (GAN), composed of a generator and discriminator, has become a hot research topic and is considered a feasible approach to solving the dehazing problems. In this study, a novel dehazed generative adversarial network (GAN) is proposed to reconstruct the clean images from the hazy ones. For the generator network of the proposed GAN, the color and luminance feature extraction module and the high-frequency feature extraction module aim to extract multi-scale features and color space characteristics, which help the network to acquire texture, color, and luminance information. Meanwhile, a color loss function based on hue saturation value (HSV) is also proposed to enhance the performance in color recovery. For the discriminator network, a parallel structure is designed to enhance the extraction of texture and background information. Synthetic and real hazy images are used to check the performance of the proposed method. The experimental results demonstrate that the performance can significantly improve the image quality with a significant increment in peak-signal-to-noise ratio (PSNR). Compared with other popular methods, the dehazing results of the proposed method closely resemble haze-free images.

## Introduction

Remote sensing technology is a popular detection technology that utilizes aircraft, satellites, or other cruise equipment to capture ground information. Notably, it can break through the limitations of terrain to monitor a wide area with acceptable cost, especially with the application of Unmanned Aerial Vehicles (UAVs)^1^. For its good performance, remote sensing has been applied in resource exploration^2^, environmental monitoring^3^, agricultural surveys^4^, and other fields^5^. However, the collected images are always affected by the natural environment. For example, water vapor and micro-particles in the air often condense to form haze floating in the atmosphere, which will interfere with the refraction of light and degrade the precision of images. Particularly, the haze phenomenon may even introduce artifacts in some conditions. Therefore, it is urgent to concentrate on developing dehazing algorithms.

To tackle the aforementioned concern, some conventional image dehazing Techniques have been developed. Generally, the conventional approaches are broadly categorized into three groups: image enhancement-based methods^6,7^, depth-of-field-based methods^8^, and prior-knowledge-based methods^9,10^. Specifically, image enhancement-based methods use conventional image enhancement techniques to reduce artifact information. Here, filtering algorithms or physical models are designed to separate the artifacts that exist in the hazy images. However, these methods will always bring unexpected feature loss of effective information, and the artifacts also cannot be accurately removed. For the depth-of-field-based methods, we often utilize sensors and other equipment to gather scene-depth information in hazy images and estimate the critical parameters by using mathematical and physical models. On this basis, we simulate the acquisition environment and dehaze images by incorporating the relevant parameters into the atmospheric scattering model. It is worth noting that obtaining an accurate estimation of modeling parameters is relatively difficult in real cases, preventing wide applications of these methods. As for prior-knowledge-based methods, they aim to construct an appropriate atmospheric scattering model by analyzing numerous hazy images and using the acquired prior knowledge to tell the clean images. Notably, the estimation process can also be viewed as constructing a statistical mapping between the hazy image and the target image. However, the prior knowledge does not always fit the real situation because the environment is variable. As we know, the reconstructed results are sensitive to the prior knowledge. It means that image distortion and the creation of new artifacts may occur with inaccurate prior information. In summary, although conventional image dehazing methods can ease the haze phenomenon and improve the image quality to some extent, they still have defects in terms of reconstruction accuracy.

Recently, many algorithms of deep-learning^11,12^ have been constructed to cope with the dehazing problems of images. Among them, convolutional neural networks (CNNs) are extensively discussed and successfully used in many applications because of their excellent properties, such as translation invariance and high-dimensional feature extraction capability. For the dehazing task, CNN-based methods^13^ can accurately fit the mapping relationship that exists between the hazy and clear images, therefore obtaining better reconstruction results and processing performance than the conventional methods. More importantly, the mapping establishment process is accomplished adaptively without manual parameter fitting procedures. Therefore, we can get the point that CNN-based methods are more suitable to dehaze the artifacts and improve the quality of remote-sensing images, compared with commonly used conventional methods.

Although the effectiveness of classical CNN-based methods is verified, their performance still needs further improvement to recover the high-frequency features and local information perfectly. Meanwhile, these frameworks also show limited performance when confronted with effective information, having similar properties to unwanted artifacts. All these shortcomings are derived from the fact that the architectures and design principles for the classical CNN-based methods are relatively simple, which shows limitations in the processing of complicated images.Therefore, Generative Adversarial Networks have been applied by scholars^14,15^ in the field of image dehazing. To obtain more perfect haze-free images, a modified generative adversarial network (GAN) framework with texture and color space enhancement is proposed. It aims to minimize the loss of texture, color, and brightness information as much as possible while removing artifacts. Besides, we propose the hue saturation value (HSV) loss function to reduce color distortion. The following are contributions:

1. We design a color and luminance feature extraction module to reduce color and luminance distortion during the haze removal process. It can extract more rich features from HSV and RGB color space, respectively.
2. We propose to initially separate texture images of remote sensing images using the Kirsch method. Subsequently, we design a high-frequency feature extraction module to capture edge feature information of texture images. It can extract more rich edge features.
3. We also design a multi-scale feature extraction module, a hybrid attention module, a detail feature extraction module, a feature fusion, and an image restoration module to extract main features and achieve haze removal in the remote sensing images.
4. We design a discriminative network to determine whether dehazed output by the generative network is a real or dehazed haze-free image. It can improve the performance of the generator. In addition, we also propose the hue saturation value loss function to combine it with other loss functions. This enables a more precise measurement of the color variance between the haze-free remote sensing image and the dehazed remote sensing image.

## Related work

Numerous methods currently utilized for image dehazing mainly align with two categories: conventional methods and deep-learning based approaches. The conventional methods mainly rely on image enhancement or prior information to dehaze images. For the conventional methods, Abbasi et al. proposed fuzzy histogram equalization^16^. It proposed a novel estimation by constructing an affiliation function. Although this method is better than the traditional histogram equalization, it cannot retain detailed information and has large color distortion. Liu et al. proposed a dehazed model based on improved Retinex^17^. It introduces multiple coefficient terms to construct a nonlinear model and proposes a variational Retinex model. Although it can handle hazy images well compared with the traditional Retinex method, it will leave some artifacts when dealing with highly thickly hazy images. Dong et al. proposed a homomorphic filter based on an improved homomorphic filter^18^. It can improve the subsequent target detection accuracy, but it appears grayscale map conversion. Agrawal et al. proposed a dehazing method using superpixel and nonlinear transformation^19^. It uses superpixel as prior knowledge, which builds a nonlinear transformation model. It forms a haze-free image after processing the brightest region and smoothing the transmission. Although this method can remove the artifacts of remote sensing images well, the information, such as color and luminance, is not well restored.

Compared with conventional methods, remote sensing image dehazing methods based on deep learning can automatically learn features from the images without manually designing complex rules and feature extractors. It offers superior dehazing effects, has broader applicability, and is more suited for practical applications. Jiang et al. proposed a deep hybrid network^20^. It consists of a haze residual attention sub-network and a refinement sub-network. Although it can recover haze images with large concentrations, it cannot recover the details of the distant view and has a large luminance and color distortion. Ma et al. proposed a temporal information injection network by introducing the time series information^21^. It can remove artifacts of images under different hazy concentrations. However, there is a loss of high-frequency detail information in the recovered images. Ma et al. also proposed a spectral grouping network for dehazing hyperspectral images^22^. Although it can dehaze images with different thick hazy, it has a loss of some contour and edge information. Zhang et al. proposed a non-local network for removing the dense haze of image^23^. It consists of full point-wise convolution and non-local loss. Although it can restore contour and texture information of dehazed images, there is luminance and color distortion in dehazed images. Yu et al. proposed a multispectral-based CNN remote sensing haze removal model^24^. This method can reduce the loss of high-frequency information while dehazing images. However, there is still some luminance distortion. Yin et al. proposed a variational image dehazing convolutional neural network^25^. It can better recover detailed information and color information. However, there are some residual artifacts in the recovered image. Kuanar et al. proposed a convolutional neural network based on DeGlow-DeHaze^26^. It uses an extended network to improve the color and luminance recovery. However, it cannot completely recover the detailed information, and there are still some artifacts in dazed images.

As one of the CNN frameworks, generative adversarial networks (GANs) can be regarded as a specific type of artificial intelligence network. The conventional artificial intelligence network only contains one network. However, GANs is composed of two integral components: the generative network, designed specifically for dehazing remote sensing images, and the adversarial network, aimed at enhancing the generative network’s efficacy. Consequently, when juxtaposed against traditional artificial intelligence networks, GANs exhibits superior performance in the dehazing process for remote sensing images. Several dehazing techniques employing GANs are proposed. Li et al. proposed feature attention GAN with fusion discriminator^27^. It is based on two-branch migration learning sub-network. Although it can effectively remove artifacts and exhibit better color restoration, dehazed image has a loss of detailed information. Chen et al. proposed GAN included a multi-stage memory-attention module and a dual region discriminator^28^. Although it can remove non-uniform haze in the image, dehazed images lose some effective information. Liu et al. proposed AMEA-GAN^29^. The structure encompasses a retinal attention mechanism dedicated to dehazing and a color enhancement module. While capable of reducing color distortion and minimizing the loss of detailed information, it lacks the ability to eliminate heavy haze in remote sensing images effectively. Liu et al. proposed spatial information fusion self-attention generative adversarial network^30^. It is based on a GAN consisting of some attention mechanisms. It can effectively remove non-uniform haze from the image and restore the real color tint. However, dehazed images lose some texture information. He et al. proposed an asymmetric contrastive CycleGAN dehazing framework^31^. It is a CycleGAN consisting of a feature transfer network. Although it can successfully remove non-uniform haze without loss of detailed information, it causes color and luminance distortion. Chen et al. proposed a depth-aware haze generation network^32^. The structure comprises an independent depth estimation network alongside GAN. Although this model is more effective in retaining effective information, there are still some artifacts in the image after dehazing. Wang et al. proposed the dehazing model of CycleGAN^33^. It consists of cyclic self-perceptual loss and the improved CycleGAN. Although it can remove artifacts of hazy images and prevent color distortion, dehazed images have a loss of detailed information. Dong et al. proposed semi-supervised GAN^34^. It consists of a domain alignment module, a haze-aware attention module, and the dark channel prior. SDA-GAN can remove artifacts in hazy images, but it leads to loss of detail information and color distortion.

Although all the above methods can dehaze the hazy images, there are problems such as detail distortion, color and luminance distortion, and residual artifacts in dehazed images. Therefore, we introduce a Generative Adversarial Network (GAN) model tailored for the dehazing of remote sensing images. This model adeptly removes haze of diverse densities, diminishes texture information loss, and corrects color and luminance distortions.

## Method

In pursuit of ameliorating the adverse impact of haze on remote sensing imagery and augmenting their fidelity, we have developed a specialized generative adversarial network designed specifically for the dehazing process of remote sensing images. The proposed network comprises a generator and discriminator. The generator is tasked with dehazing remote sensing images, whereas the discriminator’s role is to differentiate between input remote sensing images: whether they are authentic clear images or generated by the generator. This interplay between the discriminator and generator serves to bolster the dehazing proficiency of the generator specifically for remote sensing images. In the subsequent sections, individual introductions to the generator, discriminator, and loss function will be provided. Compared to existing algorithms, our generator includes a new color and luminance feature extraction module, texture information enhancement module, and feature extraction networks. The discriminator consists of two distinct modules to discern image features. The high-frequency feature extraction module consists of the Kirsch method and the high-frequency feature extraction module. It can reduce the loss of color and other information in the remote sensing images after dehazing. The texture information enhancement module can reduce edge information distortion. The new feature extraction network extracts richer information through the multi-scale feature extraction module and other modules. Therefore, our generator focuses on more image information compared to other dehazing algorithms. As for the discriminator, it includes a background and texture information feature extraction part and a global information feature extraction part. The background and texture information feature extraction section reduces the loss of feature extraction information. Compared to existing discriminator networks, the global information feature extraction part makes the network deeper to increase the receptive field. It can enhance the discriminator’s information extraction capability. Finally, we designed a feature fusion section to merge the extracted information. Therefore, our discriminator focuses on more feature information.

### Proposed generator

**Figure 1:**
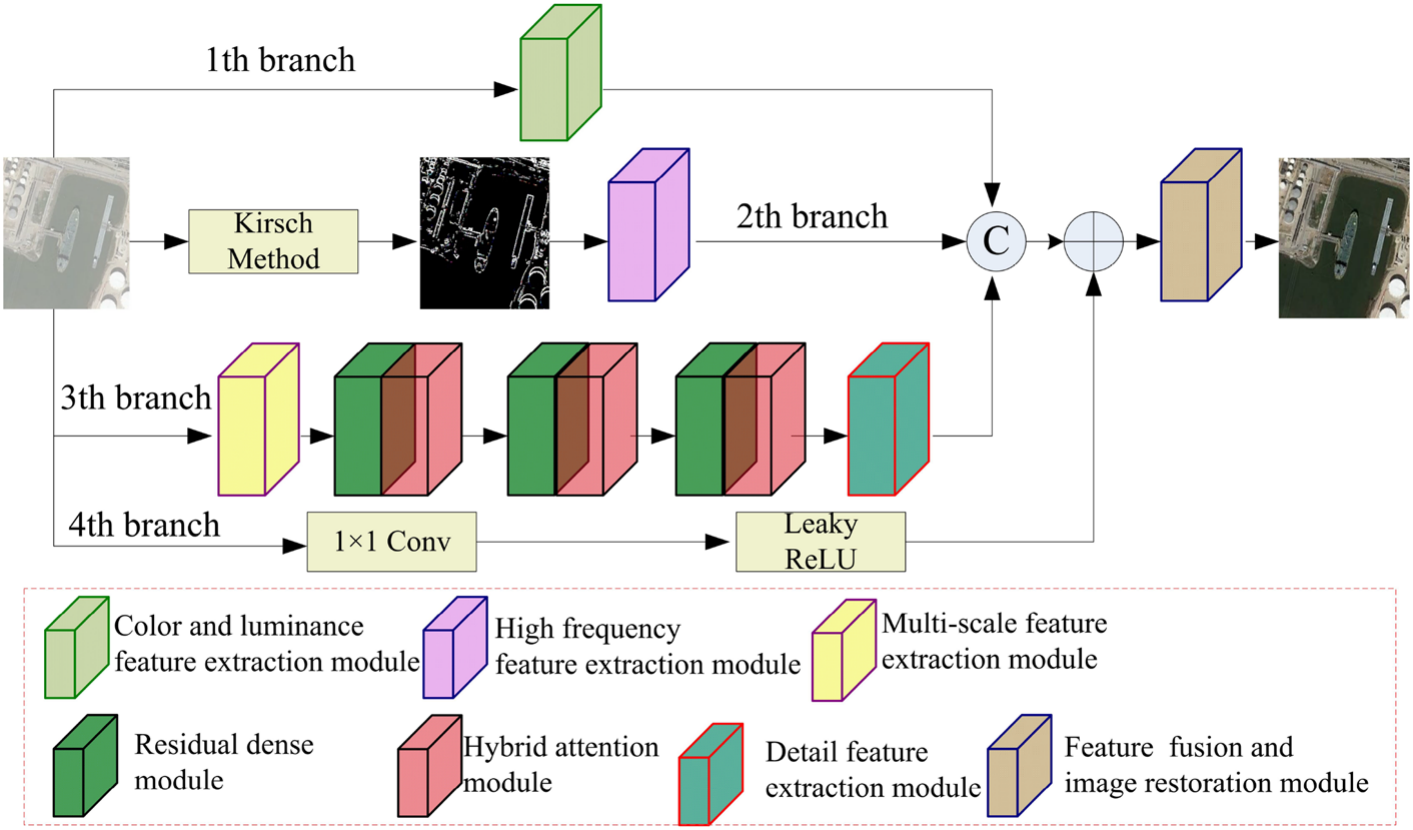
Proposed generator.

Figure 1 displays the proposed generator. It contains four branches. The first branch is mainly utilized to extract color and luminance information of remote sensing images, called the color and luminance feature extraction module. The primary function of the second branch, incorporating the Kirsch method and a high-frequency feature extraction module, is extracting texture information from remote sensing images. The third branch is the backbone of extracting features, which consists of a multi-scale feature extraction module, three residual dense modules, three hybrid attention modules and a detail feature extraction module. The fourth branch is a $1\times 1$ convolutional layer with LeakyReLU is mainly utilized to preserve shallow feature information. The concatenation unit is utilized to fuse the extracted features from the first, second, and third branches. We conduct an element-wise addition between the integrated outcome and the output derived from the fourth unit to realize the combination of shallow and depth features. In the end, a feature fusion and image restoration module has been devised to amalgamate the extracted features further and reconstruct the remote sensing image.

**Figure 2:**
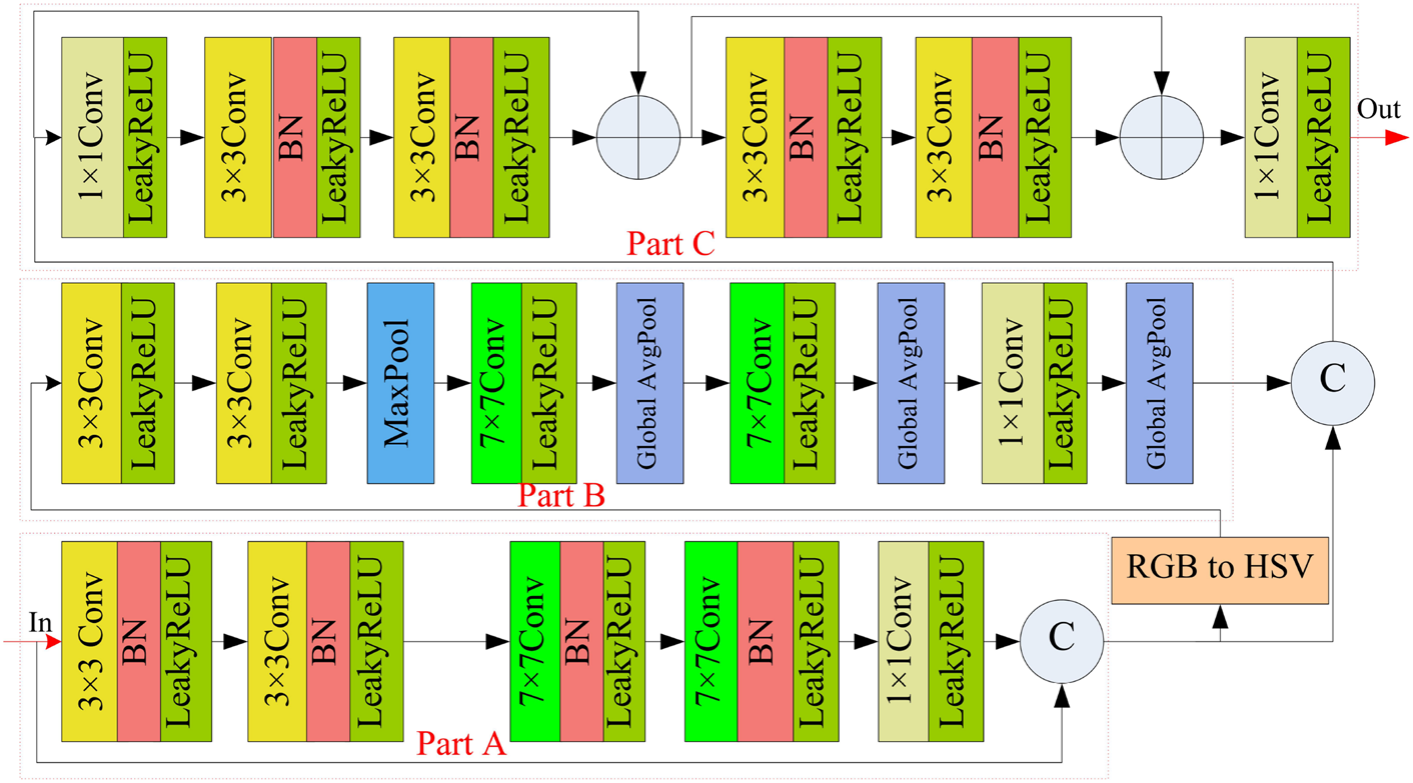
Proposed color and luminance feature extraction module.

Compared to existing algorithms, the color and luminance feature extraction module can reduce the loss of color, brightness, and other information. Firstly, it extracts the feature information of the image in the RGB color space dimension. Next, the image is converted into the HSV color space dimension. Then, the color and luminance feature extraction module extracts features from images in the HSV color space dimension using the network module. It enhances the Retention of brightness, luminance, and other information. Finally, we designed a fusion module. It will integrate two different sets of feature information.

The proposed color and luminance feature extraction module utilized in Fig. 1 is shown in Fig. 2. It consists of Part A, B, and C networks. In part A network, it utilizes double $3\times 3$ convolution layers and two $7\times 7$ convolution layers to extract the semantic information in RGB color space. Each $3\times 3$ convolution layer and $7\times 7$ convolution layer are succeeded by a batch normalization layer (BN) and a LeakyReLU activation function (LeakyReLU). In addition, a $1\times 1$ convolutional layer with LeakyReLU is utilized for adjusting the channel number of the extracted feature map by two $3\times 3$ convolution layers and $7\times 7$ convolution layers, aligning it with the channel number of the original image. To enhance the retention of valuable information, a concatenation operation is utilized to merge the primary image. The hue saturation value (HSV) color space contains three pieces of information: hue, saturation, and luminance, which can better identify the difference between artifacts of high luminance and effective information in hazy remote sensing images. Therefore, we convert the output image of part A network from the RGB color space to the HSV color space and design part B network to extract color information of the remote sensing hazy images from the HSV color space.

In part B network, it firstly uses two $3\times 3$ convolution layers with LeakyReLU for the initial extraction of features. Secondly, it uses the max pooling layer to extract important color information. Thirdly, $7\times 7$ convolution layers with LeakyReLU are employed to expand the receptive field, facilitating the extraction of broader global color information. In addition, global average pooling layers serve to enhance local color information, thereby reducing color distortion or image sharpening. Fourthly, a $1\times 1$ convolution layer augmented with LeakyReLU is positioned to regulate the channel count within the extracted feature map, ensuring parity between the output feature map’s channel counts in the part B network and that of the part A network. In the end, the features extracted from the HSV color space in part B network and those from the RGB color space in part A network are merged via a concatenation operation. The resultant fused feature map is then employed as the input for part C network.

In part C network, it firstly uses a $1\times 1$ convolution layer augmented with Leaky-ReLU to be employed to integrate fused feature information from different channels, which makes fusion among the feature information extracted from different color spaces. Further feature extraction is conducted on the fused results obtained from the feature maps extracted by networks part A and B. This process involves four modules, each consisting of a sequence comprising a $3\times 3$ convolutional operation, normalization, and application of the LeakyReLU activation function. In the end, a $1\times 1$ convolution layer with the LeakyReLU activation function is used to adjust the channel number of the output feature map, which is useful for concatenating with other modules.

**Figure 3:**
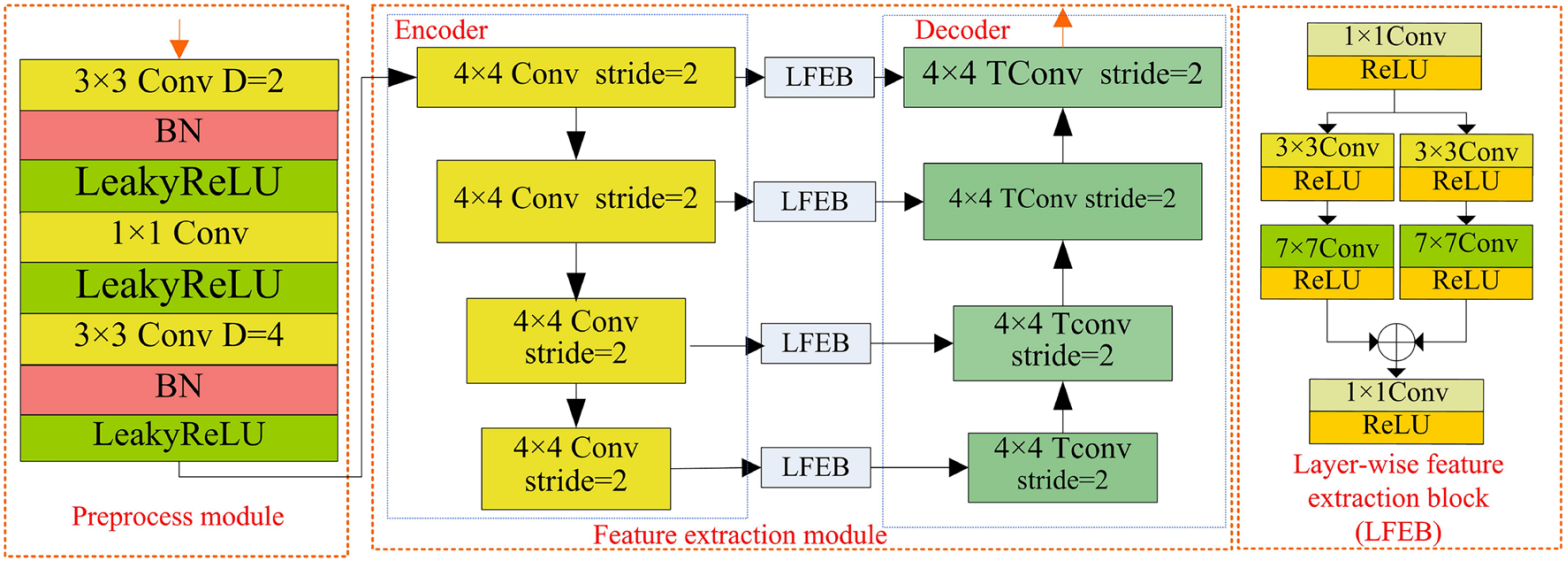
Proposed high-frequency feature extraction module.

The remote sensing image contains both low-frequency information and high-frequency information. It is difficult to directly extract high-frequency edge information by using convolutional neural networks. Compared to other algorithms, we have designed a new texture enhancement part. It includes the Kirsch physical method and a high-frequency feature extraction module. Firstly, we utilize the Kirsch physical method to separate the high-frequency information (texture image) of the image during the network feature extraction process. Then, we employ an improved high-frequency extraction module. It can extract the high-frequency information of the image for the network. We add a new preprocess part and a layer-wise feature extraction block to the existing module. The preprocessing part enables the network to extract the feature information of the texture image under a larger receptive field. The layer-wise feature extraction section connects the module’s down-sampling layer and up-sampling layer. It can reduce the loss of feature information and make the network more stable. Therefore, the Kirsch method is firstly employed within the second branch depicted in Fig. 1 to separate the texture image from the remote sensing image. Secondly, a high-frequency feature extraction module shown in Fig. 3 is proposed to capture edge feature information from the texture image. It consists of two parts: a preprocessing module and a feature extraction module.

The preprocessing module can enlarge the receptive field, which provides more global information from the texture image for the high-frequency feature extraction module. It comprises two concatenated dilated convolutional layers featuring distinct dilated rates. The two concatenated expansion convolutions will cause feature dispersion, which lead to the loss of information. To mitigate information loss, we add a $1\times 1$ convolutional layer as a connecting layer between the two dilated convolution layers. The feature extraction module shown in Fig. 3 consists of an encoder, decoder, and Layer-wise feature extraction block (LFEB). The encoder is used to extract positional information of pixels on remote sensing texture images. The encoder comprises four $4\times 4$ convolutional layers with a stride of 2, aimed at extracting features from feature maps with varying receptive fields. The output feature map size resulting from the $4\times 4$ convolutional layers with a stride of 2 is halved compared to the input feature map size. Subsequently, the decoder is employed to extract semantic edge information from the feature maps obtained through the encoder. It utilizes four $4\times 4$ transposed convolutional layers that stride is 2 to extract features from feature maps with different receptive fields. a $4\times 4$ transposed convolutional operation with a stride of 2 doubled the scale of the input feature map. To enhance the network’s focus on image edge features while minimizing the loss of shallow feature information, we design a Layer-wise feature extraction block and utilize four Layer-wise feature extraction blocks to connect four convolutions in the decoder with their corresponding transposed convolutions in the encoder. In LFEB, we firstly change the number of channels to 1 by using a $1\times 1$ convolution layer with ReLU activation function (ReLU), which aims to fuse the features along the different channels. A parallel module is employed to capture features from diverse sizes of outputs across multiple layers of the encoder. It comprises two identical branches, each comprising a $3\times 3$ convolutional layer with ReLU activation and a $7\times 7$ convolutional layer also employing ReLU activation. A $1\times 1$ convolutional layer with ReLU activation is employed to adjust the channel count.

**Figure 4:**
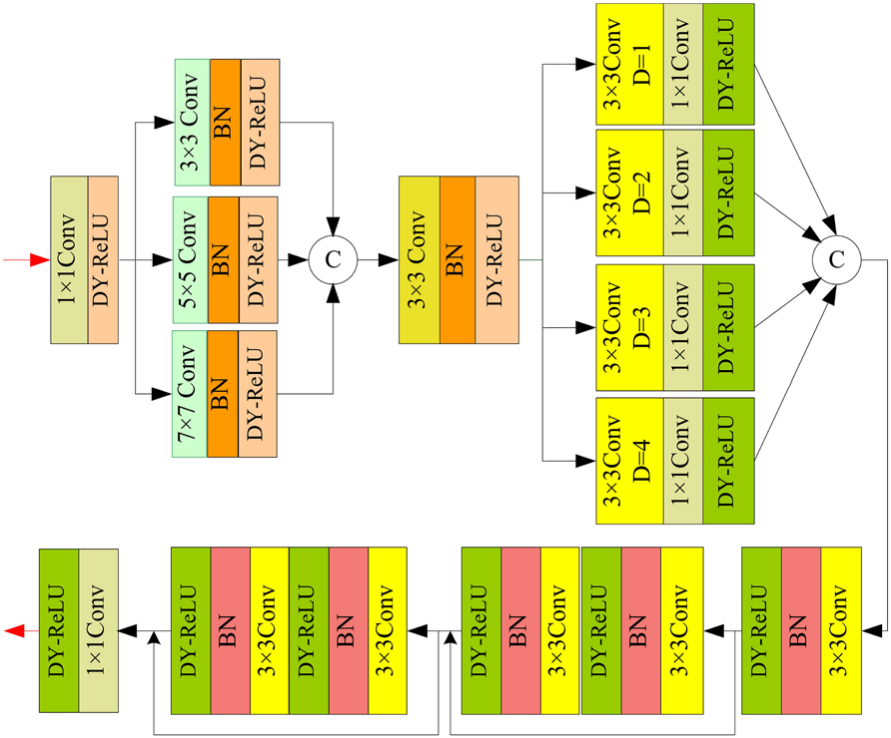
Proposed multi-scale feature extraction module.

Compared to other multi-scale modules, our multi-scale feature extraction module consists of an existing multi-scale enhancement part, a new receptive field enhancement part, and a feature fusion part. The receptive field enhancement part includes dilated convolutions with different dilation rates and convolutional layers with a kernel size of 1. It can provide the network with larger and more feature information under different receptive fields. Using multiple convolutional layers, the fusion extraction module merges the feature information obtained from different receptive fields. Therefore, compared to existing multi-scale modules, our module focuses on more feature information under different receptive fields. The added feature fusion part makes the network more stable.

The proposed multi-scale feature extraction module utilized in Fig. 1 is shown in Fig. 4. Firstly, we utilize a $1\times 1$ convolution with DY-ReLU activation function (DY-ReLU) to increase channel count. Secondly, three parallel convolutions composed of $3\times 3$, $5\times 5$, and $7\times 7$ convolutions with BN and DY-ReLU respectively, are used to capture global and detailed feature information with different scales. The utilization of a large convolutional kernel facilitates the extraction of global features, whereas employing smaller convolutional kernels serves to extract more detailed features. The features extracted by parallel convolutions are fused by concatenation operation and a $3\times 3$ convolution with BN and DY-ReLU. Thirdly, we utilize four parallel branches to capture texture feature information under multiple receptive fields. Each branch is composed of a $3\times 3$ dilated convolution, a $1\times 1$ convolution, and an activation function. The dilation rates of $3\times 3$ dilated convolutions are 1, 2, 3, and 4, respectively. The $1\times 1$ convolution layers are used to keep away from the risk of feature information loss caused by dilated convolution. Fourthly, we use two convolutional groups with residual structures to extract deep features. Each convolutional group consists of two modules. Each module consists of a $3\times 3$ onvolution, BN, and DY-ReLU. In the end, a $1\times 1$ convolutional operation is applied to adjust the channel count.

**Figure 5:**
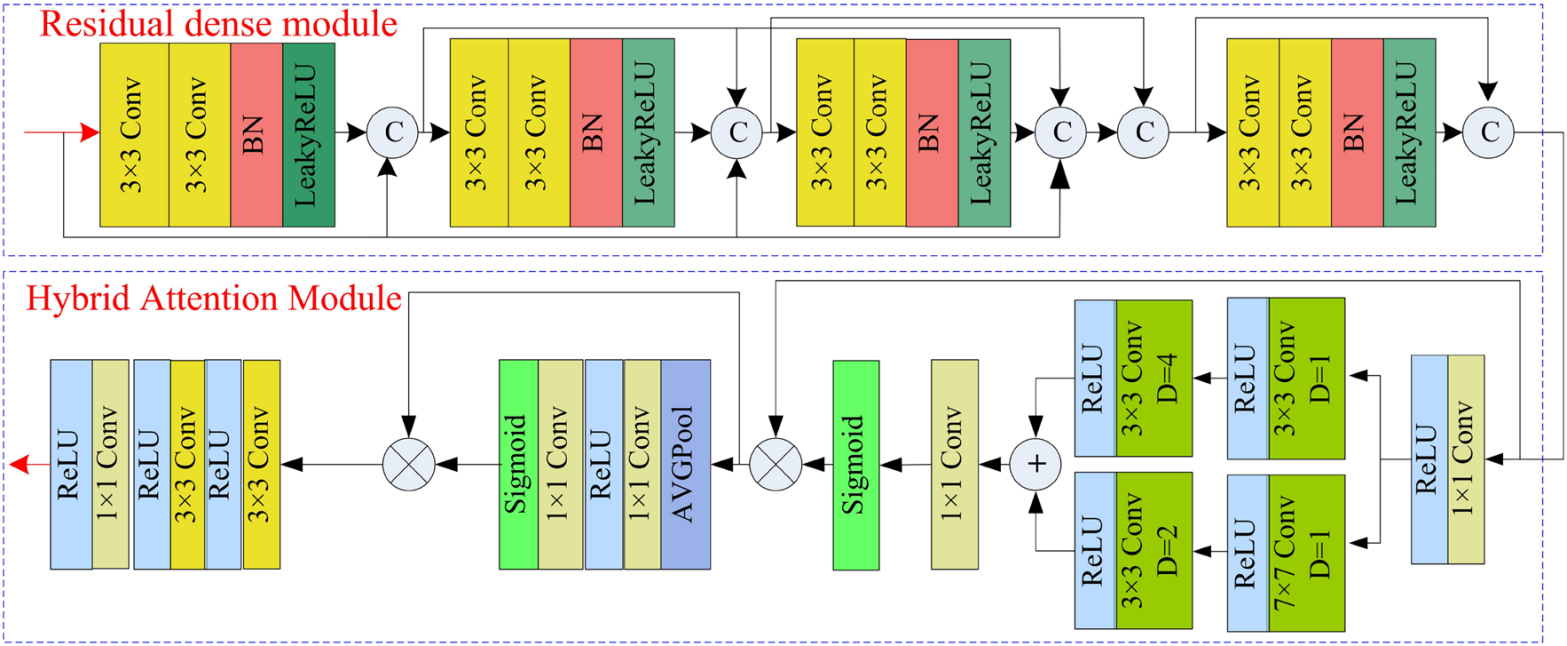
Residual dense module and hybrid attention module.

Compared to feature extraction networks in other dehazing algorithms, we designed the residual dense module and hybrid attention module. The feature extraction module combines dense layers and Residual layers. It can reduce the loss of feature information in deep networks. Dense layers increase the utilization of feature information through parameter sharing between different network layers. We added a mixed attention module to enhance the ability to extract global features.

The residual dense module and hybrid attention module utilized in Fig. 1 are shown in Fig. 5. We utilize the residual dense module to extract features. It uses two cascading $3\times 3$ convolutional layers with BN and LeakyReLU as a basic module. Four basic modules and skip connections constitute the residual dense module. The two $3\times 3$ convolutional layers enhance the capture of feature information in linear space. Additionally, the inclusion of BN and LeakyReLU layers serves to bolster the robustness and generalization of the model, augmenting its nonlinear capabilities. We incorporate skip connections within the basic module to preserve features from shallower layers of the network. To enhance the focus of the network on crucial features, we also designed a hybrid attention module connected with the residual dense module. In the hybrid attention module, the initial step involves applying a $1\times 1$ convolutional layer to adjust the feature map channels, and a parallel convolution to compute the spatial feature maps’ weight. The parallel convolution consists of two branches. One branch comprises convolution consists of two branches. One branch comprises two $3\times 3$ convolutions whose dilation rates are 1 and 4, along with ReLU. The other branch consists of a $7\times 7$ convolution whose dilation rate is 1, a $3\times 3$ convolution whose dilation rate is 2, and a ReLU activation function. Subsequently, an additional $1\times 1$ convolutional layer is employed to alter the channel count, alongside the utilization of the sigmoid function to normalize the spatial weight. The spatial attention feature map is derived by multiplying the spatial weights with the input feature map. Secondly, to derive the channel weights, we employ an average pooling layer and two $1\times 1$ convolutions for down-sampling and up-sampling in the channel dimension, respectively. The second sigmoid function is also utilized to normalize the channel weight. The channel weights multiplied by the input spatial attention feature map result in the final feature map of the hybrid attention. In the end, we utilize two $3\times 3$ convolution to further capture features and $1\times 1$ to adjust the channel number.

**Figure 6:**
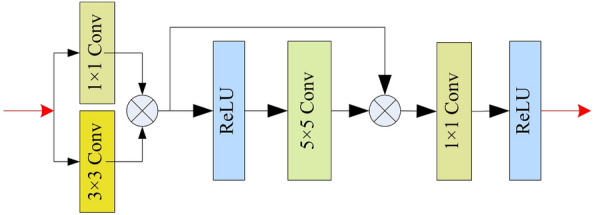
Proposed detail feature extraction module.

Compared to existing algorithms, the detail extraction module we designed performs element-wise multiplication of feature information at different scales. It enables the network to learn more about the spatial relationships between pixels in the image. The proposed detail feature extraction module utilized in Fig. 1 is shown in Fig. 6. We firstly use parallel $1\times 1$ convolution and $3\times 3$ convolution as a basic unit to capture detail features, and element-wise multiply the features extracted from the two branches to achieve feature enhancement. In addition, a $5\times 5$ convolution with residual structure is utilized to increase the receptive field to obtain global features. In the end, the secondary $1\times 1$ convolutional operation is employed to modify the channel count, and the ReLU activation function is utilized to augment the module’s nonlinear capabilities.

**Figure 7:**
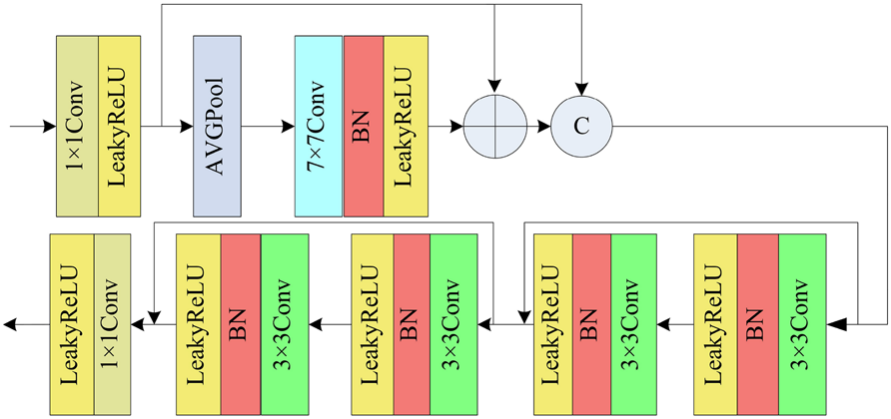
Proposed feature fusion and image restoration module.

The proposed fusion and image restoration module utilized in Fig. 1 is shown in Fig. 7. Firstly, a $1\times 1$ convolutional operation is applied to regulate the channel count and amalgamate the concatenated feature map. Secondly, we utilize a global average pooling, a $7\times 7$ convolution, element-wise addition, and concatenate operation to extract features from the fused feature maps. The element-wise addition operation can reduce the gradient vanishing, and the concatenate operation can reduce the loss of information. Thirdly, two mixed modules with residual structure are used to restore the remote sensing image. There are two convolution layers in each mixed module. Each convolution layer is comprised of a $3\times 3$ convolution, BN, and LeakyReLU. In the end, a $1\times 1$ convolutional layer is employed to regulate the channel count to 3. Compared to existing algorithms, we have designed a fusion module tasked with integrating different feature information. These include features extracted by the network, additional high-frequency feature information, and additional feature information from different color space dimensions. Our fusion module can reduce distortion in the fused feature information. The residual edges in the generator help reduce the loss of image feature information and prevent gradient vanishing.

#### Proposed discriminator

**Figure 8:**
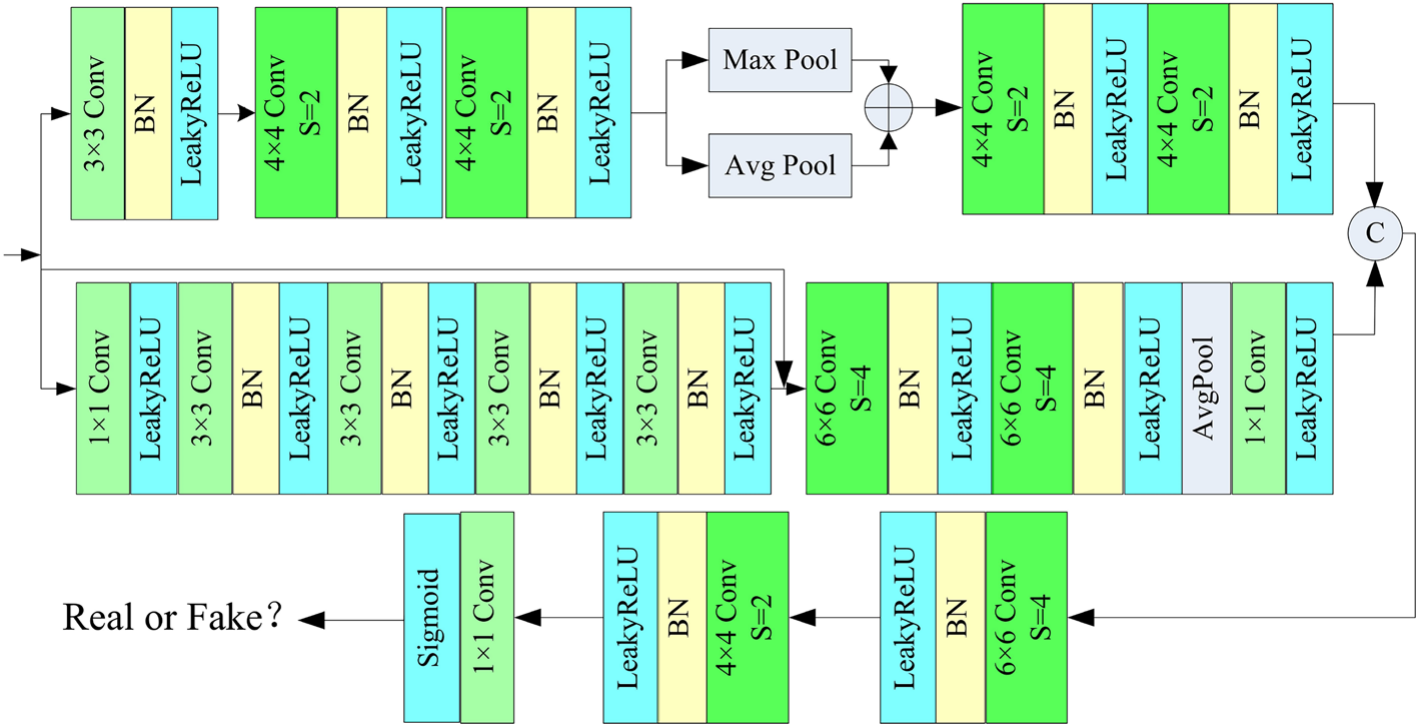
Proposed discriminator.

The discriminative network is chiefly utilized to differentiate whether the input remote sensing image is a dehazed version or a real high-resolution image. The input to the adversarial network comes from the output of the generative network and actual haze-free images. It can help enhance the dehazing capability of the generative network for remote sensing images. Ideally, the adversarial network should not be able to differentiate whether the generated output image is a dehazed remote sensing image or a real, haze-free remote sensing image. Our design discriminator is shown in Fig. 8, which consists of two offshoots. In the upper offshoot, we firstly use a $3\times 3$ convolution to extract features and adjust the number of channel. Secondly, Two $4\times 4$ convolutional operations with a stride of 2 are employed to diminish the size of the feature map and extract more detailed information. Thirdly, we use parallel average pooling and max pooling to extract background information and texture information, respectively, from hazy remote sensing images. In the end, two $4\times 4$ strided convolutions with stride = 2 are used to extract more depth information. In the lower offshoot, we firstly use four cascaded $3\times 3$ convolutions and a skip connection to capture detail features and expand receptive field. Secondly, We employ two $6\times 6$ strided convolutions with a stride of 4 to decrease the feature map size and extract global information, and average pooling to enhance background information. Thirdly, we use concatenation operation to fuse the output feature maps obtained from the two offshoots. In the end, the size of the feature map is reduced to 1 by using a $6\times 6$ strided convolution with stride = 4 and $4\times 4$ strided convolution with stride = 2. Sigmoid function is utilized for normalizing the result of the adversarial network to output discrimination scores.

Compared to other existing discriminator networks, our discriminator consists of three parts: background and detail feature extraction, deep network global feature extraction, and multi-scale fusion downsampling. Firstly, the background and detail feature extraction part can extract richer semantic information of the image background and texture information. The background information includes environmental information where actual objects in the image are located. The texture information includes edge information of the image. Therefore, the discriminator can focus on more information between different objects and the background contained in the positions of the pixels. Secondly, the deep network global feature extraction part extracts the global information of the image through multiple convolutional layers. It enlarges the discriminator’s receptive field by using multiple downsampling convolutional layers. It also enhances the feature representation ability. Finally, we designed a multi-scale concatenated fusion module. It can fuse background information, detail information, and global information under different scales of the receptive field. The multi-scale downsampling layers can also provide the discriminator with feature information of different levels of detail. The added information fusion part can help the discriminator learn the relationship between different feature information. It also enhances the network’s ability to learn the spatial relationships between image pixels. Therefore, our designed discriminator can focus on richer image feature information compared to existing discriminators. It can also reduce pixel distortion caused by excessive feature information.

#### Loss function

To make the dehazed remote sensing images generated by the generative network more closely resemble real haze-free remote sensing images, we propose the improved adversarial network loss function by proposing hue saturation value (HSV) loss. The loss function in our method is:

1 $$\begin{aligned} Loss = {L_{adv}}+ {L_1} + {L_{HSV}} \end{aligned}$$

where $\ L_{adv}$ is the adversarial loss. It is an important part of the GAN to realize the adversarial training of generator and discriminator. It is defined as follows:

2 $$\begin{aligned} {L_{adv}} = E[\log D(y)] + E\{ \log [1 - D(G(x))]\} \end{aligned}$$

where $\ x$ signifies the input hazy remote sensing image, $\ y$ represents the haze-free remote sensing image generated by the generator. $\ E[ \bullet ]$ represents the calculation of the mean. $\ D$ denotes the adversarial network, and $\ G$ symbolizes the generator network. $\ {L_1}$ is employed to quantify the disparity between the haze-free remote sensing image and the dehazed remote sensing image. It is defined as follows:

3 $$\begin{aligned} {L_1} = E[|y - G(x)|] \end{aligned}$$

In order to more accurately gauge the color distinction between the authentic haze-free remote sensing image and the generated image, we proposed $\ {L_{HSV}}$ that is expressed as following:

4 $$\begin{aligned} {L_{HSV}}(y,f({x_{hsv}})) = E\left[ 1 - \frac{{(2{\beta _y}{\beta _{f({x_{hsv}})}} + {\varepsilon _1}) + (2{\eta _{yf({x_{hsv}})}} + {\varepsilon _2})}}{{(\beta _y^2 + \beta _{f({x_{hsv}})}^2 + {\varepsilon _1})(\eta _y^2 + \eta _{f({x_{hsv}})}^2 + {\varepsilon _2})}}\right] \end{aligned}$$

where $\ {{x_{hsv}}}$ denotes the image in the HSV color space, $\ \beta$ denotes the mean and $\ \eta$ denotes the standard deviation, $\ {{\varepsilon _1}}$ and $\ {{\varepsilon _2}}$ are penalty terms added to increase network stability. They are set to 0.0001 and 0.0009 respectively. Additionally, $\ y$ represents the genuine haze-free image, while $\ f({x_{hsv}})$ denotes the haze-free image generated by the network.

## Experiment

To validate the efficacy of the proposed method alongside other exemplary approaches for dehazing remote sensing images. We compare our method with Cycle-SNSPGAN^33^, RefineD-Net^35^, SDA-GAN^34^, and PSMB-Net^36^ on NWPU-RESISC45 dataset^37^. The dataset comprises 31,500 haze-free remote sensing images distributed across 45 categories. Each scene category encompasses 700 remote sensing images, all sized at $256\times 256$ pixels. To construct the training dataset for model training, we generate hazy remote sensing images based on the haze-free remote sensing images using the atmospheric scattering model. The atmospheric scattering model is defined as:

5 $$\begin{aligned} l(x) = J(x)t(x) + {A_\infty }(1 - t(x)) \end{aligned}$$

where $\ l(x)$ represents the synthesized hazy remote sensing image. $\ J(x)$ denotes the haze-free remote sensing image. $\ {A_\infty }$ signifies the value of atmospheric light at infinity. $\ t(x)$ denotes the transmittance and is expressed as:

6 $$\begin{aligned} t(x) = \exp ( - \beta \times d(x)) \end{aligned}$$

where $\ \beta$ represents atmospheric scattering factor, $\ d(x)$ represents the atmospheric light intensity. In our experiment, the transmittance is set to [0.05, 0.2] and the light intensity is set to 1.

We generated 31,500 synthesized hazy remote sensing images using the atmospheric scattering model. Subsequently, we paired each haze-free remote sensing image with its corresponding hazy remote sensing image to form image pairs. We randomly select 25,200 paired images as the training dataset and 6300 paired images as the validation dataset. To assess the performance of various methodologies quantitatively, we use the peak signal-to-noise ratio (PSNR), structural similarity index (SSIM), and mean squared error (MSE) to measure the difference between the haze-free remote sensing image and hazy remote sensing image. In our experiments, all methods use the same parameters. The patch size is $256\times 256$. The epoch is 200 and the learning rate is 0.0001. The batch size is 1. Our experiments use the PyTorch deep learning platform based on the Ubuntu 18.04 experimental platform, and the model training is performed on NVIDA 3090Ti GPU. The training parameters are shown in Table 1.

**Table 1:** Training parameters of our method.

Training parameters	Specifications
Patch size	$256\times 256$
Batch size	1
Epoch number	200
Learning rate	0.0001
Optimizer	Adam

### Simulation on the synthesized images

**Figure 9:**
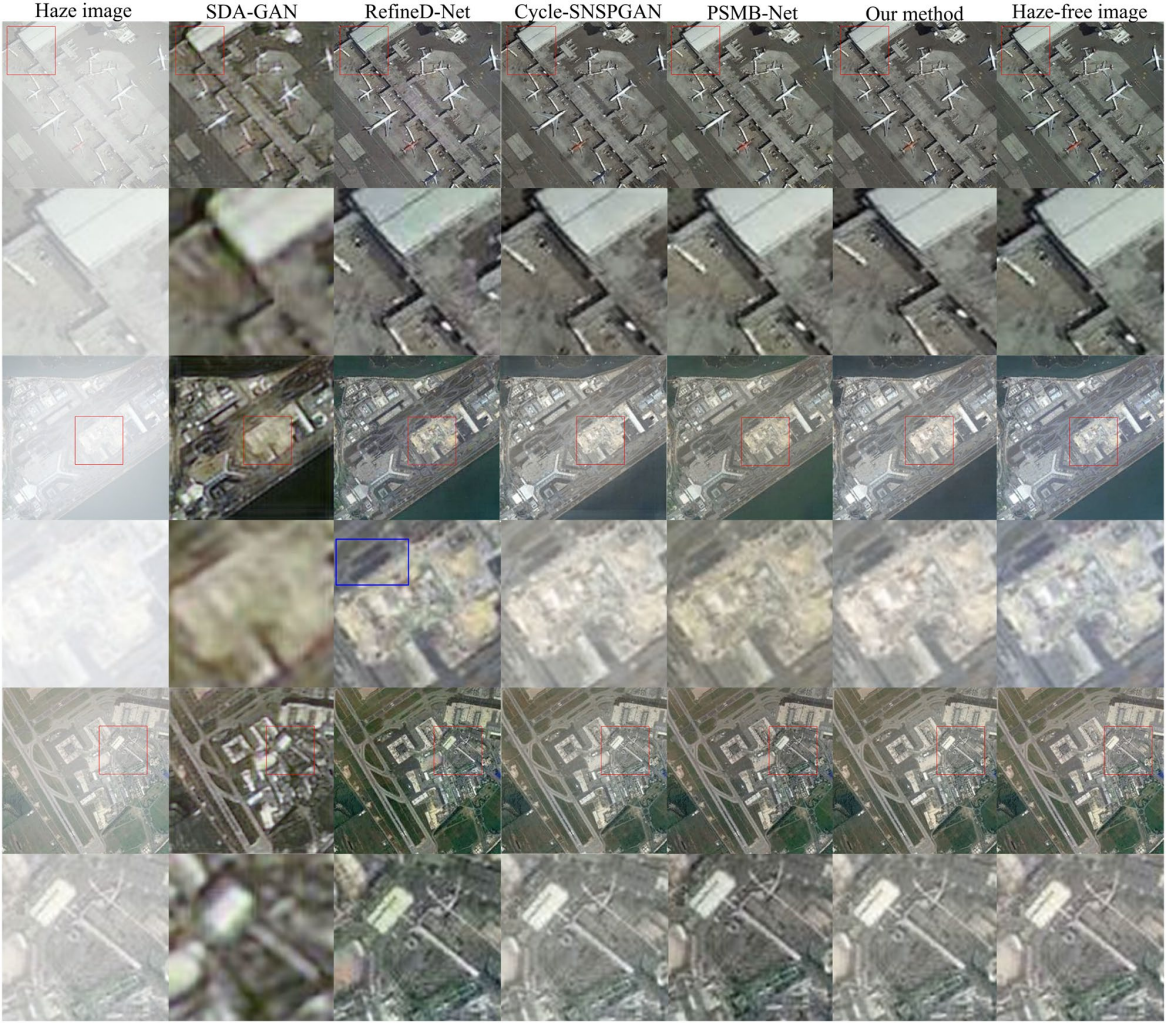
Dehazing results for different synthetic hazy remote sensing images.

**Table 2:** The dehazing performance of five methods.

Metrics	SDAGAN	RefineD-Net	Cycle-SNSPGAN	PSMB-Net	Our method
PSNR	23.1736	26.9639	29.2607	30.2963	35.3553
SSIM	0.7737	0.9564	0.9665	0.9766	0.9826
MSE	0.0096	0.0039	0.0047	0.0019	0.0008

**Figure 10:**
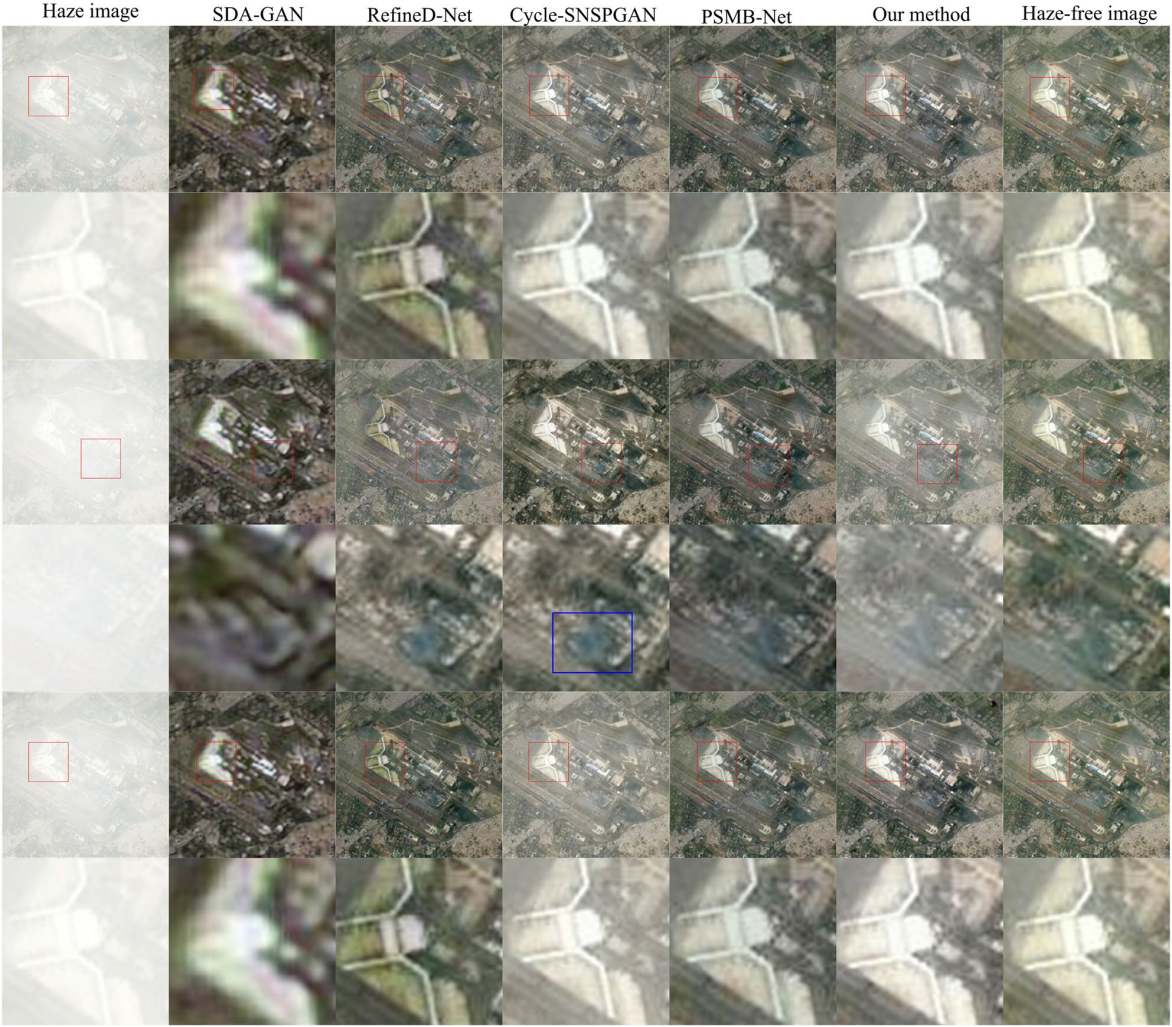
Dehazing results for hazy remote sensing with different thickness of haze.

**Table 3:** The dehazing performance of five methods.

Metrics	Haze-thickness	SDAGAN	RefineD-Net	Cycle-SNSPGAN	PSMB-Net	Our method
PSNR	0.1	23.4250	27.1100	29.2607	33.1940	37.1952
	0.12	17.1880	26.3006	29.2838	27.0204	34.6560
	0.15	17.0830	21.6722	25.2032	24.2327	30.1841
	Avg	19.2320	25.0276	25.2673	28.1490	34.0118
SSIM	0.1	0.7045	0.9544	0.9665	0.9864	0.9925
	0.12	0.6923	0.9418	0.9394	0.9878	0.9882
	0.15	0.6970	0.9605	0.9708	0.9718	0.9768
	Avg	0.6979	0.9523	0.9589	0.9820	0.9858
MSE	0.1	0.0181	0.0077	0.0047	0.0019	0.0001
	0.12	0.0191	0.0093	0.0047	0.0019	0.0003
	0.15	0.0195	0.0068	0.0030	0.0097	0.0009
	Avg	0.0189	0.0079	0.0041	0.0025	0.0004

Three randomly selected paired remote sensing images from the validation dataset are depicted in Fig. 9, with the hazy remote sensing image displayed in the first column and the haze-free remote sensing image showcased in the last column. The images in columns 2 to 6 are the dehazed images obtained by SDA-GAN, RefineD-Net, Cycle-SNSPGAN, PSMB-Net, and our method, respectively.

The images in the first, third, and fifth rows displayed in Fig. 9 are complete images. Meanwhile, the images in the second, fourth, and sixth rows represent locally magnified portions of their respective complete image. In the second row, it shows that SDA-GAN loses some of the edge and detail information, resulting in lower clarity in locally magnified images. The image clarity of SDA-GAN is the lowest, followed by Re-fineD-Net. The image clarity of other methods is higher, and it is difficult to distinguish differences with the naked eye. In the fourth row, the image clarity of SDA-GAN is the lowest, followed by RefineD-Net. There is a noticeable yellow tint in the images generated by SDA-GAN, RefineD-Net, and PSMB-Net. The SDA-GAN exhibits significant color distortion, followed by PSMB-Net and RefineD-Net. Although the image clarity of Cycle-SNSPGAN and our method is close, Cycle-SNSPGAN exhibits some loss of detail. The lost details are annotated with blue boxes in the locally magnified image. In the sixth row, the image clarity of SDA-GAN is the lowest, followed by RefineD-Net. The luminance of the image generated by PSMB-Net is distorted. The color of the image generated by RefineD-Net is distorted. The images produced by our method exhibit a closer resemblance to the haze-free remote sensing images compared to those generated by other methods.

For a quantitative assessment of various methods, comprehensive tests were conducted utilizing all images from the test dataset for each method. The tabulated results are presented in Table 2. The PSNR values of SDA-GAN, RefineD-Net, Cycle-SNSPGAN, PSMB-Net, and our method are 23.1736, 26.9639, 29.2607, and 30.2963, respectively. The SSIM values of SDA-GAN, RefineD-Net, Cycle-SNSPGAN, PSMB-Net, and our method are 0.7737, 0.9564, 0.9665, 0.9766 and 0.9826, respectively. The MSE values of SDA-GAN, RefineD-Net, Cycle-SNSPGAN, PSMB-Net, and our method are 0.0096, 0.0039, 0.0047, 0.0019, and 0.0008, respectively. As depicted in Table 1, our method showcases the highest PSNR and SSIM scores, coupled with the lowest MSE. These outcomes substantiate that our method exhibits a superior capacity to eliminate haze from remote sensing images when compared to other methods.

**Table 4:** Training parameters of our method.

Metrics	SDAGAN	RefineD-Net	Cycle-SNSPGAN	PSMB-Net	Our method
FLOPs(G)	48.073	29.495	134.291	208.708	1520.996
Parameters(M)	9.960	65.759	9.432	17.711	48.502

To validate the performance of different methods across various levels of haze, we randomly selected one clear remote sensing image and generated three hazy remote sensing images by introducing distinct levels of haze. The hazy remote sensing image is shown in the first column in Fig. 10. The images in columns 2 to 6 are the dehazed images obtained by SDA-GAN, RefineD-Net, Cycle-SNSPGAN, PSMB-Net, and our method, respectively. The final column is the haze-free remote sensing images. The images in the first, third, and fifth rows displayed in Fig. 10 are complete images. Meanwhile, the images in the second, fourth, and sixth rows represent locally magnified portions of their respective complete image. In the second row, the remote sensing image clarity of SDA-GAN is lower. The image generated by RefineD-Net introduces a greenish tint, signifying color distortion. It appears that the image generated by Cycle-SNSPGAN is overexposed, while that produced by PSMB-Net is underexposed. In the fourth row, it is obvious that the images generated by SDA-GAN, RefineD-Net, and PSMB-Net differ significantly from the haze-free image. Although the images generated by our method and Cycle-SNSPGAN are closer to the haze-free remote sensing image, the image generated by Cycle-SNSPGAN still has some distortion that has been marked with the blue box. In the sixth row, it is evident that the locally magnified image generated by SDA-GAN is relatively blurry. The locally magnified images produced by RefineD-Net display noticeable color distortion. Additionally, images generated by Cycle-SNSPGAN exhibit signs of overexposure, while those generated by PSMB-Net suffer from issues of underexposure. Although there are some distortions in dehazed images as the haze density increases, the dehazed images of our method maintain a closer semblance to authentic haze-free remote sensing images.

The quantitative analysis results are shown in Table 3. As the haze thickness in remote sensing images increases, the PSNR and SSIM values decrease, and the MSE values increase for all methods. However, our method consistently maintains the highest PSNR and SSIM values, along with the lowest MSE values. This demonstrates that our proposed method still has effective dehazing capability for hazy remote sensing images with different thicknesses of haze.

We use a randomly selected remote sensing image with a size of $256\times 256$ to discuss the complexity of each model. Table 4 lists the FLOPS and model parameter metrics for SDA-GAN, RefineD-Net, Cycle-SNSPGAN, PSMB-Net, and our method. The FLOPS values of SDA-GAN, RefineD-Net, Cycle-SNSPGAN, PSMB-Net, and our method are respectively 48.073, 29.495, 134.291, 208.708, and 1520.996. The model parameter values of SDA-GAN, RefineD-Net, Cycle-SNSPGAN, PSMB-Net, and our method are 9.960, 65.759, 9.432, 17.711, and 48.502, respectively. The experimental results indicate that our model has a higher complexity compared to existing models, but it is tolerable considering the dehazing effect.

#### Simulation on the real images

**Figure 11:**
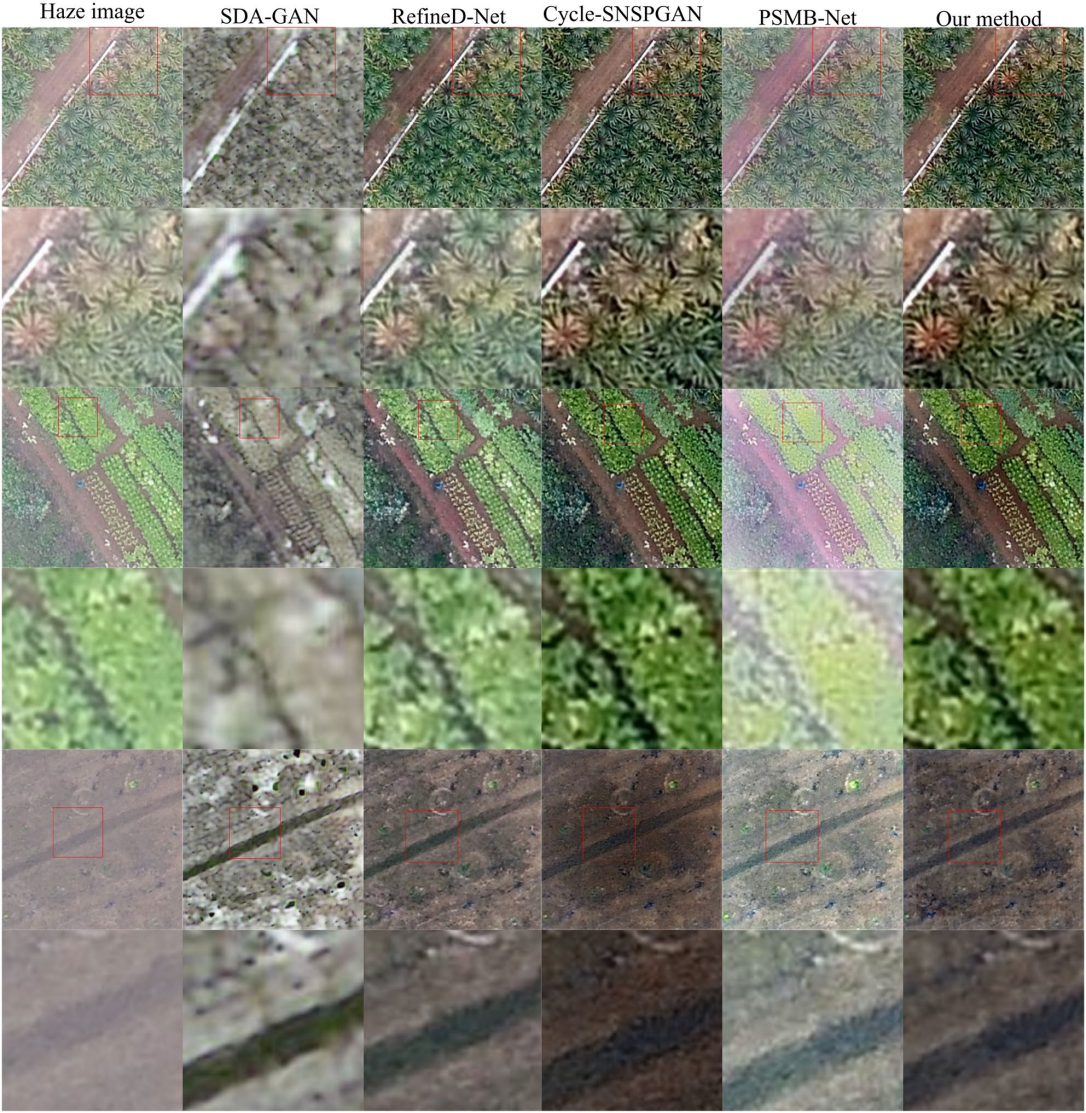
Dehazing results of experimental models in real remote sensing images.

**Figure 12:**
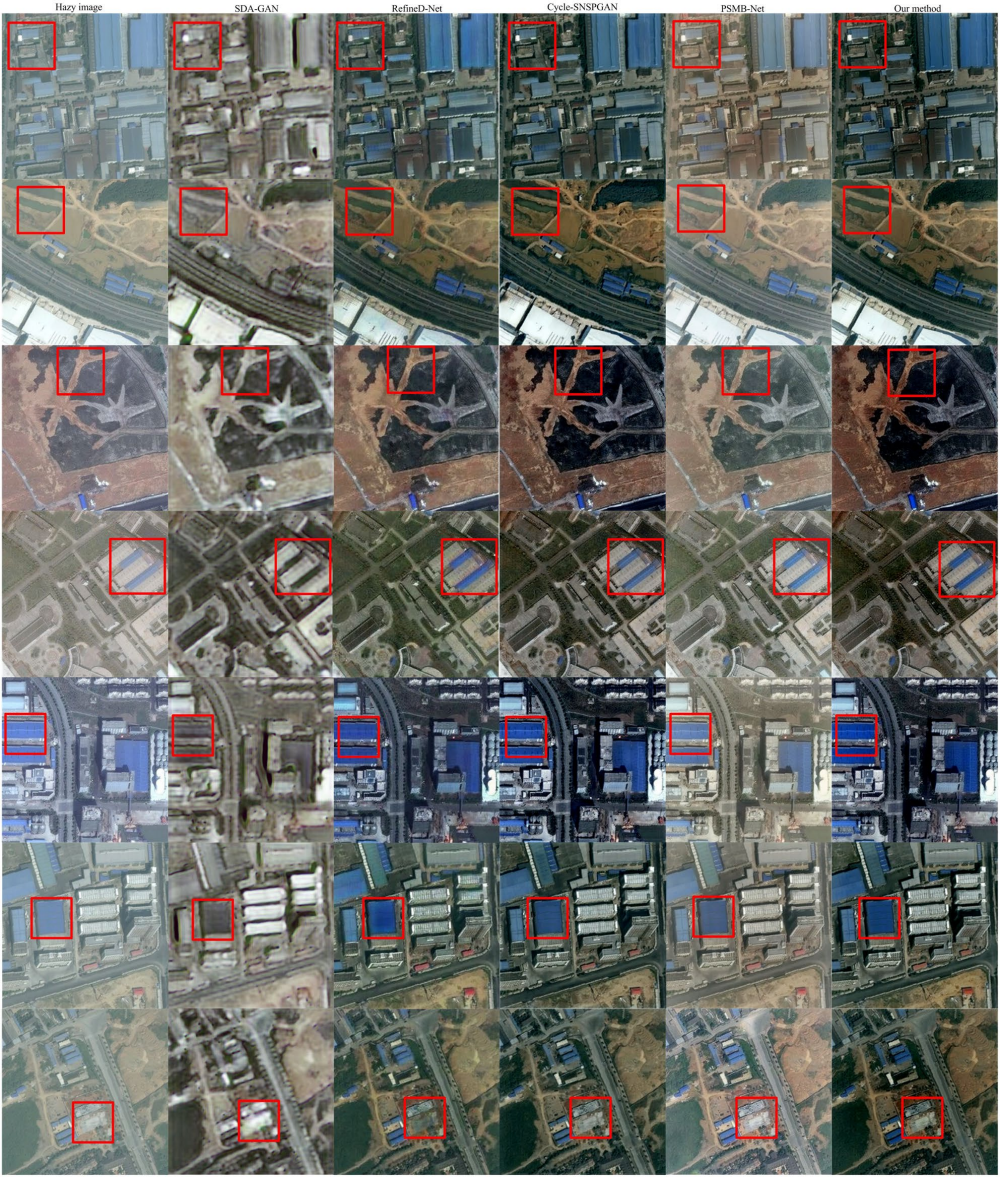
Dehazing results of experimental models in real remote sensing images from various scenarios.

To assess the dehazing capabilities of various dehazing methods on authentic remote sensing images, three hazy remote sensing images^38^ in the real world were chosen for processing. The first row in Fig. 11 is the real hazy remote sensing images and its locally magnified images. The second row to the sixth row are the dehazed remote sensing images and their locally magnified images generated by SDA-GAN, RefineD-Net, Cycle-SNSPGAN, PSMB-Net, and our method, respectively. In the first row, images generated by SDA-GAN have poor clarity and exhibit severe distortions. The image generated by PSMB-Net contains a significant amount of artifacts. The locally magnified image generated by RefineD-Net in the second row is overexposed, leading to higher luminance. The images produced by both Cycle-SNSPGAN and our method display heightened clarity. In the third row, the image generated by SDA-GAN continues to demonstrate pronounced distortion. The image generated by PSMB-Net still contains a considerable amount of haze and exhibits noticeable color distortion. The image generated by RefineD-Net has noticeable artifacts in the images, leading to image blurring. The images produced by both Cycle-SNSPGAN and our method still display heightened clarity. In the fifth row, the image generated by SDA-GAN shows both color distortion and blurring. The images generated by RefineD-Net, Cycle-SNSPGAN, and PSMB-Net exhibit color distortion. The edge distortion also manifests in the images generated by RefineD-Net. Considering the above analysis, the images that our method generated have higher clarity and better visual perception.

We have included the outcomes of dehazing real hazy remote sensing images from various scenarios. As shown in Fig. 12, the first column of images represents real remote sensing hazy images. The second to sixth columns depict the dehazing results of SDA-Net, RefineD-Net, Cycle-SNSPGAN, PSMB-Net, and our method, respectively. In the first row, the dehazing result of SDA-Net exhibits edge blurring. The outcomes of RefineD-Net and PSMB-Net both show color distortion. There are a small number of artifacts in the image (mark in the red-framed area). The dehazing result of Cycle-SNSPGAN displays color deviation. In the second row, the result of SDA-Net shows low image clarity. The outcome of RefineD-Net has some artifacts along the edges. The portion within the red frame exhibits lower clarity. As for Cycle-SNSPGAN, the part of the image marked by the red frame appears relatively blurred. The image generated by PSMB-Net contains plenty of artifacts. In the fourth row, the result of SDA-Net exhibits color distortion within the red-framed area. The image generated by RefineD-Net is blurry in the red-framed area. Both the images generated by Cycle-SNSPGAN and PSMB-Net show color deviation within the red-framed area. In the fifth row, the result of SDA-Net is blurred. The image generated by RefineD-Net has a low clarity within the red-framed area. There is color distortion within the red-framed area in the result generated by Cycle-SNSPGAN. The result of PSMB-Net is overexposed. In the sixth row, the result of SDA-Net exhibits color information loss within the red-framed area. The image generated by RefineD-Net is blurred within the red-framed area. The results from Cycle-SNSPGAN and PSMB-Net have lighter colors and reduced brightness. In the seventh row, the result of SDA-Net exhibits very low clarity. The outcomes’ quality of RefineD-Net and Cycle-SNSPGAN is within the red-framed area. The result from PSMB-Net is overexposed within the red-framed area. Based on the above analysis, the images generated by our model demonstrate better color preservation and higher clarity.

#### Simulation on RICE1 dataset

The RICE1 dataset contains 500 pairs of remote sensing images sized $512\times 512$. The experimental data is displayed in Table 5. The PSNR values of SDA-GAN, RefineD-Net, Cycle-SNSPGAN, PSMB-Net, and our method are 28.2746, 29.1317, 30.3350, 32.0430, and 35.5193, respectively. The SSIM values of SDA-GAN, RefineD-Net, Cycle-SNSPGAN, PSMB-Net, and our method are 0.6583, 0.8258, 0.9494, 0.9436, and 0.9774, respectively. The MSE values of SDA-GAN, RefineD-Net, Cycle-SNSPGAN, PSMB-Net, and our method are 0.0045, 0.0038, 0.0023, 0.0017, and 0.0009, respectively. Our method achieved the highest PSNR and SSIM values, as well as the lowest MSE value.

**Table 5:** The dehazing performance of five methods in RICE1 dataset.

Metrics	SDAGAN	RefineD-Net	Cycle-SNSPGAN	PSMB-Net	Our method
PSNR	28.2746	29.1317	30.3350	32.0430	35.5193
SSIM	0.6583	0.8258	0.9494	0.9436	0.9774
MSE	0.0045	0.0038	0.0023	0.0017	0.0009

**Figure 13:**
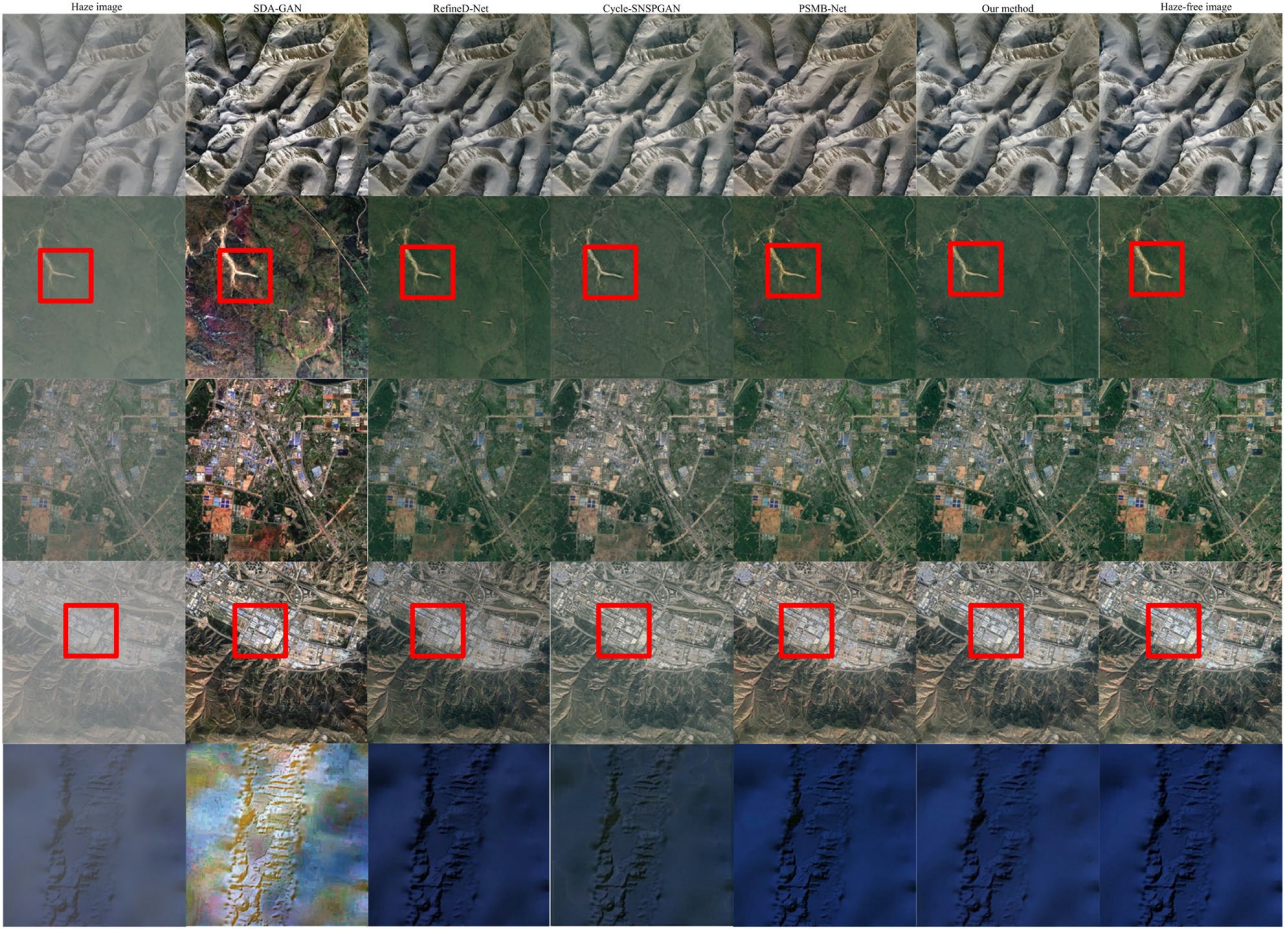
Dehazing results of experimental models in RICE1 dataset.

We randomly selected five dehazed images. The experimental results are shown in Fig. 13. The first column depicts the hazy images. The second to sixth columns represent the outputs from SDA-GAN, RefineD-Net, Cycle-SNSPGAN, PSMB-Net, and our dehazing method respectively. The seventh column shows the corresponding haze-free images. In the first row, the result from SDA-GAN exhibits high exposure. The outcomes of RefineD-Net and PSMB-Net have color deviations. Cycle-SNSPGAN introduces greenish bias in its result. In the second row, there is the color deviation in the result of SDA-GAN. The result of RefineD-Net has low brightness. The outcome of Cycle-SNSPGAN exhibits light colors. PSMB-Net’s result shows high similarity to the real images compared to the image generated by our model. In the third row, SDA-GAN’s result introduces red color bias. The outcome of RefineD-Net has low clarity. The resultCycle-SNSPGAN is high exposure. The image generated by PSMB-Net has low brightness. In the fourth row, there is the color deviation in the results of both SDA-GAN and Cycle-SNSPGAN. The outcome of RefineD-Net is low clarity. The image generated by PSMB-Net exhibits high exposure. In the fifth row, the result of SDA-GAN introduces green and white biases. RefineD-Net’s outcome is deep color. The output of Cycle-SNSPGAN exhibits low brightness and color deviations. PSMB-Net and our method are close to the real image. Based on the above analysis, our proposed method exhibits excellent performance in the RICE1 dataset.

#### Simulation on RICE2 dataset

The RICE2 dataset comprises 736 pairs of paired cloudy and cloud-free remote sensing images, along with 736 cloud mask images. We evaluated the cloud removal capabilities of various models on this dataset. Experimental data are presented in Table 6. The PSNR values of SDA-GAN, RefineD-Net, Cycle-SNSPGAN, PSMB-Net, and our method are 28.0674, 29.2975, 30.3810, 32.0096, and 35.1090, respectively. The SSIM values of SDA-GAN, RefineD-Net, Cycle-SNSPGAN, PSMB-Net, and our method are 0.3935, 0.6085, 0.8179, 0.8560, and 0.8764, respectively. The MSE values of SDA-GAN, RefineD-Net, Cycle-SNSPGAN, PSMB-Net, and our method are 0.0048, 0.0041, 0.0027, 0.0019, and 0.0010, respectively.

**Table 6:** The dehazing performance of five methods in RICE2 dataset.

Metrics	SDAGAN	RefineD-Net	Cycle-SNSPGAN	PSMB-Net	Our method
PSNR	28.0647	29.2975	30.3810	32.0096	35.1090
SSIM	0.3935	0.6085	0.8179	0.8560	0.8764
MSE	0.0048	0.0041	0.0027	0.0019	0.0010

**Figure 14:**
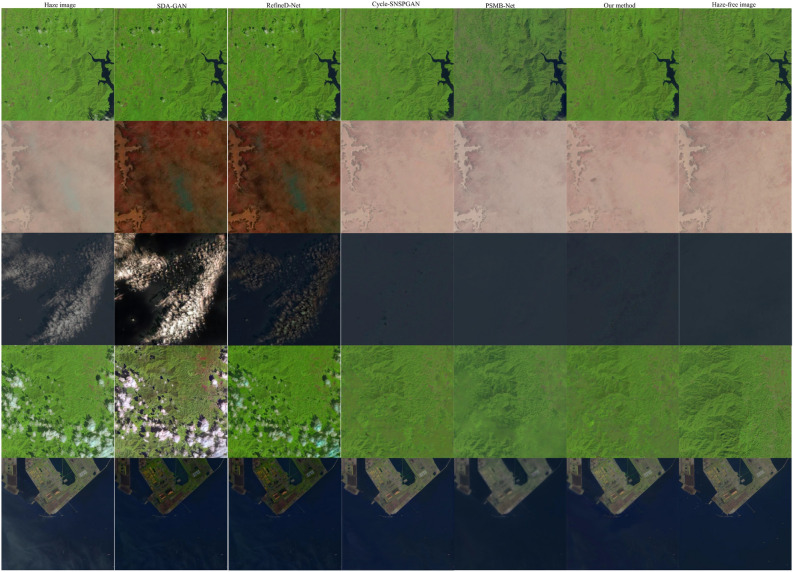
Dehazing results of experimental models in RICE2 dataset.

Figure 14 shows randomly sampled cloud removal results from the RICE2 dataset. The first column represents the cloudy images. The second to sixth columns depict the declouding results of SDA-GAN, RefineD-Net, Cycle-SNSPGAN, PSMB-Net, and our method. The seventh column represents the cloud-free images. In the first row, the results of SDA-GAN and RefineD-Net retain cloud. Cycle-SNSPGAN’s outcome introduces artifacts. The output of PSMB-Net exhibits color deviation. In the second row, the results of SDA-GAN and RefineD-Net introduce red color bias, and the clouds remain. The image generated by Cycle-SNSPGAN has lower clarity. PSMB-Net’s outcome exhibits high exposure. In the third row, the result from SDA-GAN contains many clouds. RefineD-Net’s results have a small cloud. There are remnants of artifacts as a result of Cycle-SNSPGAN. The outputs of PSMB-Net and our method are similar to cloud-free images. In the fourth row, the result of SDA-GAN introduces new cloud formations. RefineD-Net output contains small clouds. The images generated by Cycle-SNSPGAN have low clarity. The outcome of PSMB-Net exhibits color deviation. In the fifth row, there is the color deviation in the results of both SDA-GAN and RefineD-Net. The outcome of Cycle-SNSPGAN exhibits high exposure. The image generated by PSMB-Net has lower clarity. The experimental results above indicate that our method performs excellently on the RICE2 dataset.

#### Comparison of texture image results

**Figure 15:**
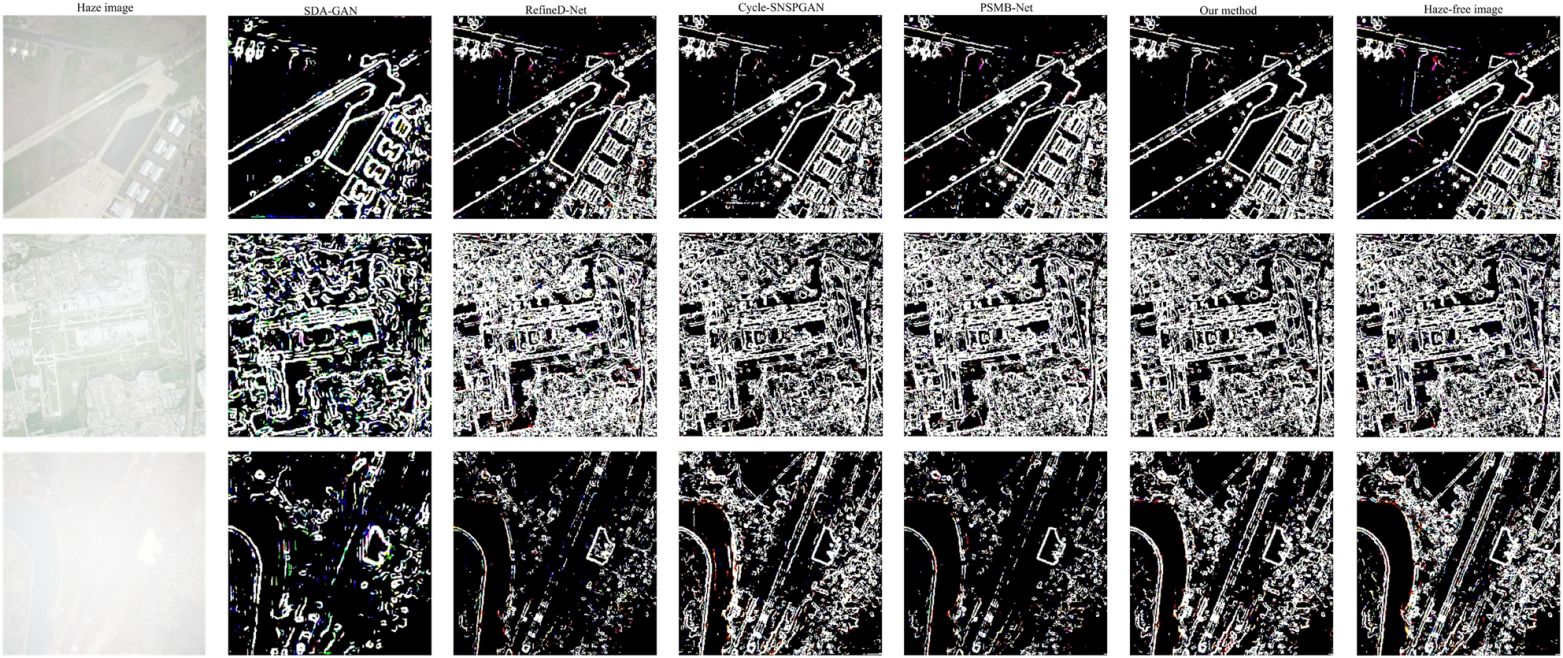
Texture images after dehazing for the five models.

The experimental results of three randomly selected images are shown in Fig. 15. The first column displays the hazy images. The second to sixth columns depict the texture images after dehazing using SDA-GAN, RefineD-Net, Cycle-SNSPGAN, PSMB-Net, and our method, respectively. The seventh column shows the texture of haze-free images. In the first row, the texture lines in the result of SDA-GAN are incorrect. The texture lines in the result of RefineD-Net are incomplete. Cycle-SNSPGAN introduces excessive texture lines in the results. PSMB-Net introduces redundant dot-like texture information in the result. In the second row, the result of SDA-GAN lacks texture lines. The texture lines in the result of RefineD-Net are not clear. Both Cycle-SNSPGAN and PSMB-Net introduce small texture lines in the results. In the third row, the results of the other four models are missing many texture lines. Our method generates texture images that are closer to the haze-free images. Table 7 displays the experimental metrics of texture images generated by five methods. The PSNR values of SDA-GAN, RefineD-Net, Cycle-SNSPGAN, PSMB-Net, and our method are 7.7376, 13.0290, 15.1812, 15.1865, and 16.5307, respectively. The SSIM values of SDA-GAN, RefineD-Net, Cycle-SNSPGAN, PSMB-Net, and our method are 0.4074, 0.7680, 0.8379, 0.8293, and 0.8752, respectively. The MSE values of SDA-GAN, RefineD-Net, Cycle-SNSPGAN, PSMB-Net, and our method are 0.1816, 0.0569, 0.0360, 0.0405, and 0.0279, respectively. Based on the above analysis, our model demonstrates excellent texture information restoration capability.

**Table 7:** Texture preservation performance of five methods.

Metrics	SDAGAN	RefineD-Net	Cycle-SNSPGAN	PSMB-Net	Our method
PSNR	7.7376	13.0290	15.1812	15.1865	16.5307
SSIM	0.4074	0.7680	0.8379	0.8293	0.8752
MSE	0.1816	0.0569	0.0360	0.0405	0.0279

#### Ablation study

To validate color and luminance feature extraction module, high-frequency feature extraction module and HSV loss function performance, an ablation experiment is designed. The result is shown in Table 8. In Table 8, the No-CLFEM means that our complete method is without color and luminance feature extraction module. The No-HFFEM means that our complete method is without a high-frequency feature extraction module. The No-PDM means that our complete method is without a parallel discriminator module. The No-HSV Loss means that our complete methods without HSV loss function.

**Table 8:** The dehazing performance of five methods.

Metrics	No-CLFEM	No-HFFEM	No-PDM	No-HSV loss	Our complete method
PSNR	29.9853	29.6919	34.0390	35.1193	35.3553
SSIM	0.9659	0.9623	0.9655	0.9768	0.9826
MSE	0.0021	0.0020	0.0026	0.0017	0.0008

The color and luminance feature extraction module is designed to extract more valuable color and luminance features. This is beneficial in minimizing color and luminance distortion. Compared to the complete method, No-CLFEM results in a reduction of 15$\%$ and 1$\%$ in PSNR and SSIM, respectively. The MSE increases by 162$\%$. This validates the effectiveness of our proposed color and luminance feature extraction module.

The high-frequency feature extraction module is designed to extract more useful texture information. This is beneficial in minimizing edge distortion. Compared to the complete method, No-HFFEM results in a reduction of 16$\%$ and 2$\%$ in PSNR and SSIM, respectively. The MSE increases by 150$\%$. This validates the effectiveness of our proposed high-frequency feature extraction module.

We proposed a parallel discriminator module to reduce the loss of texture and background information in the network. Compared to the complete method, No-PDM results in a reduction of 3$\%$ and 1$\%$ in PSNR and SSIM, respectively. The MSE increases by 225$\%$. This validates the effectiveness of our proposed HSV loss function.

We proposed HSV loss function to better more effectively quantify the color disparity between hazy and haze-free remote sensing images. Compared to the complete method, No-HSV Loss results in a reduction of 0.6$\%$ and 0.5$\%$ in PSNR and SSIM, respectively. The MSE increases by 112$\%$. This validates the effectiveness of our proposed HSV loss function.

## Conclusion

We proposed a generative adversarial network comprising a generator focused on texture, color, and luminance enhancement. It is able to remove the artifacts in remote sensing hazy images and decrease texture and color loss. Meanwhile, to empower the network to produce high-quality dehazed images, we design a parallel discriminator to improve the performance of our method. Additionally, we proposed a hue saturation value loss function to decrease color distortion. We conducted simulations under various conditions and compared our method’s results with SDA-GAN, RefineD-Net, Cycle-SNSPGAN, and PSMB-Net in generating haze-free images. Compared to other methods, the images generated by our method attained the highest PSNR and SSIM scores, along with the lowest MSE. Therefore, our proposed network stands out among existing models and demonstrates strong performance of dehazing remote sensing images. In the end, we performed ablation experiments to verify the effectiveness of the color and luminance feature extraction module, high-frequency feature extraction module, and hue saturation value loss function. The ablation experiment results demonstrate that our proposed modules and loss functions noticeably enhance the dehazing capabilities of our method. In summary, our method demonstrates strong competitiveness among existing dehazing algorithms.

## Data Availability

NWPU-RESISC45 dataset can be obtained through the website http://www.escience.cn/people/junweihan/nwpu-resisc45.html. Our authentic remote sensing data comes from Real-outdoor-UAV-remote-sensing-hazy-dataset.
